# Medicare and Medicaid Plan Integration Among Dual-Eligible Individuals

**DOI:** 10.1001/jamanetworkopen.2025.22774

**Published:** 2025-07-24

**Authors:** Hyunjee Kim, Angela Senders, Maanyatha Cheekati, Sara Edelstein, Stephan R. Lindner, Jeah Jung

**Affiliations:** 1 Center for Health Systems Effectiveness, Oregon Health & Science University, Portland; 2 Department of Health Administration and Policy, College of Public Health, George Mason University, Fairfax, Virginia

## Abstract

This cross-sectional study examines enrollee and plan-level integration among individuals eligible for both Medicaid and Medicare, with a focus on the provision of long-term care and behavioral health services.

## Introduction

Dual-eligible individuals experience fragmented care due to the complexity of navigating 2 systems. In response, Centers for Medicare & Medicaid Services (CMS) has promoted integrated plans where individuals receive both Medicare and Medicaid benefits administered by a single parent company,^1^ with the expectation of improving care coordination. Starting in 2025, CMS required exclusive plan-level integration, also known as exclusive alignment, for fully integrated dual-eligible special needs plans (FIDE-SNPs). Under this requirement, all FIDE-SNP enrollees must have Medicare and Medicaid benefits administered by the same parent company.^2^

While CMS has mandated exclusive plan-level integration for FIDE-SNPs, dual-eligible individuals in other Medicare Advantage (MA) plan types may already receive Medicare and Medicaid benefits through plans under the same parent company, even if their plans are not exclusively integrated. This enrollee-level integration could impact care coordination and outcomes, yet most research has examined only plan-level integration. In 2022, 78.1% of FIDE-SNP enrollees and 6.2% of highly integrated dual-eligible special needs plan (HIDE-SNP) enrollees were also in CMS-designated applicable integrated plans—exclusively integrated plans that meet additional criteria.^3^ However, the prevalence of enrollee-level integration and its association with plan-level integration across MA plan types remains unknown.

This study fills this gap by examining both enrollee-level and plan-level integration, with a specific focus on whether Medicaid managed care organizations under the same parent company as the MA plan covered long-term care and/or behavioral health services. These services are critical to meeting the complex needs of dual-eligible individuals and are, therefore, essential for understanding the scope of integration.^4^

## Methods

This cross-sectional study included dual-eligible individuals in standard MA plans, coordination-only dual-eligible special needs plans (D-SNPs), HIDE-SNPs, and FIDE-SNPs in December 2021. We determined enrollee-level integration by comparing the parent companies of each individual’s MA and Medicaid plan (eAppendix in Supplement 1). The study was conducted in accordance with STROBE reporting guidelines. The Oregon Health & Science University institutional review board approved this study with a waiver of informed consent.

We conducted 2 descriptive analyses. First, we calculated the prevalence of enrollee-level integration of Medicaid long-term care and/or behavioral health coverage by MA plan type. Second, among those with enrollee-level integration, we assessed their distribution across plans with varying degrees of plan-level integration. We categorized plans by the percentage of dual-eligible enrollees with enrollee-level integration: low (<50%), medium (50% to <75%), high (75% to <95%), or exclusive (95% to 100%).

## Results

Among 3 092 172 dual-eligible individuals, 459 496 (14.9%) had both long-term care and behavioral health integrated with their MA plan, 267 744 (8.7%) had only behavioral health integrated, and 54 371 (1.8%) had only long-term care integrated (Table 1). Integration with both services was most common among FIDE-SNP enrollees (222 174 of 264 443 enrollees [84.0%]), followed by those in HIDE-SNPs (108 412 of 733 909 enrollees [14.8%]), coordination-only D-SNPs (90 801 of 1 224 347 enrollees [7.4%]), and MA plans (38 109 of 869 473 enrollees [4.4%]). Among those with both services integrated, enrollee distribution across plans with low, medium, high, and exclusive integration differed by plan type (Table 2). In total, 206 382 FIDE-SNP enrollees (92.9%) were in highly or exclusively integrated plans, compared with 27 892 HIDE-SNP enrollees (25.7%). Most MA and coordination-only D-SNP enrollees were in plans with low-level or medium-level integration.

**Table 1.** Enrollee-Level Integration Between Medicare Advantage Plan and Medicaid Long-Term Care and Behavioral Health Plan by MA Plan Type, December 2021^a^

MA plan type	Total No. of enrollees	Enrollees, No. (%)			
		Not integrated^b^	Care integrated		
			Only long term^c^	Only behavioral health^d^	Both long-term care and behavioral health
Standard MA plans^e^	869 473	810 306 (93.2)	3751 (0.4)	17 307 (2.0)	38 109 (4.4)
Coordination-only D-SNPs	1 224 347	1 103 428 (90.1)	7179 (0.6)	22 939 (1.9)	90 801 (7.4)
HIDE-SNPs	733 909	383 280 (52.2)	17 930 (2.4)	224 287 (30.6)	108 412 (14.8)
FIDE-SNPs^f^	264 443	13 547 (5.1)	25 511 (9.6)	3211 (1.2)	222 174 (84.0)
All plans	3 092 172	2 310 561 (74.7)	54 371 (1.8)	267 744 (8.7)	459 496 (14.9)

Abbreviations: D-SNP, dual-eligible special needs plan; FIDE-SNP, fully integrated dual-eligible special needs plan; HIDE-SNP, highly integrated dual-eligible special needs plan; MA, Medicare Advantage; SNP, special needs plan.

^a^ We excluded 6 states (Arkansas, Idaho, Indiana, North Dakota, Ohio, and Virginia) due to data quality issues with Medicaid plan identifiers. We also excluded plans with fewer than 100 enrollees in December 2021.

^b^ Individuals may have integrated comprehensive medical care but do not have integrated long-term care or behavioral health coverage; dual-eligible individuals who were not enrolled in Medicaid managed care plans for long-term care or behavioral health services—for any reason, including living in states with limited or no managed care options—were classified as having a nonintegrated plan.

^c^ Includes integrated comprehensive managed care organization with long-term care coverage or a separate integrated long-term care plan.

^d^ Includes integrated comprehensive managed care organization with behavioral health coverage or a separate integrated behavioral health plan.

^e^ Does not include SNPs.

^f^ In 2021, FIDE-SNPs were required to cover Medicaid primary and acute care and long-term care services. Beginning in 2025, their required Medicaid coverage expanded to include behavioral health, home health, medical equipment, and Medicare cost sharing. Notably, FIDE-SNPs are eligible for a frailty adjustment in payments, which incentivizes FIDE-SNPs to enroll frail individuals who need long-term care services.

**Table 2.** Degree of Plan-Level Integration Among Individuals With Integrated Long-Term Care and Behavioral Health Coverage by Medicare Advantage Plan Type, December 2021^a^

MA plan type	No. of MA plans with both long-term care and behavioral health care integrated	MA plans, No. (%)			
		Low-level integration	Medium-level integration	High-level integration	Exclusive integration^b^
Standard MA plans^c^	38 109	29 463 (77.3)	8646 (22.7)	0	0
Coordination-only D-SNPs	90 801	40 767 (44.9)	44 698 (49.2)	5336 (5.9)	0
HIDE-SNPs	108 412	25 365 (23.4)	55 155 (50.9)	19 884 (18.3)	8008 (7.4)
FIDE-SNPs	222 174	15 600 (7.0)	192 (0.1)	3640 (1.6)	202 742 (91.3)
All plans	459 496	111 195 (24.2)	108 691 (23.7)	28 860 (6.3)	210 750 (45.9)

Abbreviations: D-SNP, dual-eligible special needs plan; FIDE-SNP, fully integrated dual-eligible special needs plan; HIDE-SNP, highly integrated dual-eligible special needs plan; MA, Medicare Advantage; SNP, special needs plan.

^a^ We excluded 6 states (Arkansas, Idaho, Indiana, North Dakota, Ohio, and Virginia) due to data quality issues with Medicaid plan identifiers. We also excluded plans with fewer than 100 enrollees in December 2021. This table is restricted to enrollees with both long-term care and behavioral health care coverage integrated. For these enrollees, we calculated the percentage of individuals in their Medicare plan that also had integrated long-term care and behavioral health coverage. We then categorized each enrollee’s Medicare plan by the percentage of dual-eligible enrollees with enrollee-level integration, as defined in the Methods section.

^b^ We defined exclusive as 95% to 100%, instead of 100%, because the Centers for Medicaid & Medicare Services (CMS) permits a short delay in Medicaid managed care plan enrollment for dually eligible individuals enrolling in integrated D-SNPs, as long as enrollee-level integration is completed as soon as possible thereafter. Plans meeting exclusive integration criteria in our study were all categorized by CMS as applicable integration plans in 2021. However, our definition of exclusive integration differs from CMS’s applicable integration plans designation, as the latter requires plans to meet more stringent criteria such as using unified procedures for appeals and grievances.

^c^ Does not include SNPs.

## Discussion

This cross-sectional study found that enrollee-level integration was highest in FIDE-SNPs, with most enrollees in exclusively integrated plans. In contrast, enrollees in other plan types were less likely to have integration with Medicaid services and were often in plans with low-level or medium-level integration. These findings suggest that FIDE-SNPs may be particularly well-positioned to coordinate care for dual-eligible individuals.

However, achieving both enrollee-level and plan-level integration depends on state policy, as integration is feasible only in states that deliver Medicaid services through managed care organizations. Managed care organizations’ coverage of behavioral health and long-term care varies across states.^5,6^ One limitation of our study is that integration patterns may have changed since December 2021. Further research is needed to determine whether enrollee-level and plan-level integration enhances care and outcomes, which could guide Medicaid policy and support effective care for dual-eligible populations.

## Notes

Supplement 1. **eAppendix. **Supplemental methods
eReference

Supplement 2.
Data Sharing Statement
