# Supplementary material for: Medicare and Medicaid Plan Integration Among Dual-Eligible Individuals
Source: JAMA Netw Open. 2025 Jul 24;8(7):e2522774. doi: 10.1001/jamanetworkopen.2025.22774 (PMC12551786; doi:10.1001/jamanetworkopen.2025.22774)
Supplement: Supplement 1. — eAppendix. Supplemental methods eReference [file jamanetwopen-e2522774-s001.pdf]

Supplemental Online Content

Kim H, Senders A, Cheekati M, Edelstein S, Lindner SR, Jung J. Medicare and Medicaid plan integration among dual-eligible individuals. *JAMA Netw Open.* 2025;8(7):e2522774. doi:10.1001/jamanetworkopen.2025.22774

eAppendix. Supplemental methods

eReference

This supplemental material has been provided by the authors to give readers additional information about their work.

© 2025 Kim H et al. *JAMA Network Open.*

eAppendix. Supplemental methods

eSection: Enroll-level integration by comparing Medicare Advantage and Medicaid plan.

Identification of Medicare Advantage plan's parent organization

We used the December Part C Contract Number (PTC_CNTRCT_ID_12) and Part C Plan Benefit Package (PTC_PBP_ID_12) from the 2020 Medicare Beneficiary Summary File to link to the plan's PARENT_ORGANIZATION in the 2020 Medicare Plan Characteristics File.

Identification of Medicaid plan's parent organization

We used the 16 managed care plan ID variables for December (MC_PLAN_ID_01_12 - MC_PLAN_ID_16_12) from the 2020 TAF Demographic and Eligibility File to identify all managed care plans in which an individual was enrolled. We used the MC_PLAN_IDs to link to each managed care plan name (MC_PLAN_NAME) and submitting state (STATE_CD) in the TAF Annual Managed Care Plan (APL) File. We then used the plan name and state to identify each plan's parent organization in the CMS Managed Care Enrollment by Program and Plan Report (CMS Report).[1] Parent organizations from the CMS Report were manually reviewed and revised when in error.

Identification of Medicaid plan type coverage

Medicaid managed care plans can provide different types of coverage, including comprehensive medical care, long-term services and supports, and/or behavioral health care, among other services. Both the CMS Report and the TAF APL File provide the plan type coverage, but they sometimes contain conflicting information. We generally found the CMS Report's plan type to be more accurate than TAF's, thus when plan type was available from both sources, we used the CMS Report's plan type (PROGRAM_NAME). If plan type could not be determined from the CMS Report, we used the TAF's plan type (MC_PLAN_TYPE_CD).

eReference

1. Centers for Medicare and Medicaid. Medicaid Managed Care Enrollment Report | Enrollment Data by Program and Plan. Accessed January 14, 2025. https://www.medicaid.gov/medicaid/managed-care/enrollment-report/index.html

© 2025 Kim H et al. *JAMA Network Open.*
