# Supplementary material for: Medicare and Medicaid Plan Integration Among Dual-Eligible Individuals
Source: JAMA Netw Open. 2025 Jul 24;8(7):e2522774. doi: 10.1001/jamanetworkopen.2025.22774 (PMC12551786; doi:10.1001/jamanetworkopen.2025.22774)
Supplement: Supplement 2. — Data Sharing Statement [file jamanetwopen-e2522774-s002.pdf]

Data Sharing Statement

Kim. Medicare and Medicaid Plan Integration Among Dual-Eligible Individuals. *JAMA Netw Open*. Published July 24, 2025. doi:10.1001/jamanetworkopen.2025.22774

Data
Data available: No

Additional Information
Explanation for why data not available: We used CMS claims database.
